# Supplementary material for: A multi-technique approach to understanding delithiation damage in LiCoO2 thin films
Source: Sci Rep. 2021 Jun 8;11:12027. doi: 10.1038/s41598-021-91051-3 (PMC8187655; doi:10.1038/s41598-021-91051-3)
Supplement: Supplementary file 1 — Supplementary Information 1. [file 41598_2021_91051_MOESM1_ESM.docx]

**A multi-technique approach to understanding delithiation damage in LiCoO_2_ thin films**

**Supplementary Information**

E. Salagre^1^, S. Quílez^1^, R. de Benito^1^, M. Jaafar^1,2^, H. P. van der Meulen^3,4^, E. Vasco^5^, R. Cid^5,6,7^, E. J. Fuller,^8^ A.A. Talin^8^, P. Segovia^1,2,4^, E.G. Michel^1,2,4^, and C. Polop^1,2,4*^

^1^ Dpto. Física Materia Condensada, Univ. Autónoma de Madrid, Spain

^2^ IFIMAC (Condensed Matter Physics Center), Univ. Autónoma de Madrid, Spain

^3^ Dpto. Física de Materiales, Univ. Autónoma de Madrid, Spain

^4^ Instituto Universitario de Ciencia de Materiales Nicolás Cabrera, Univ. Autónoma de

Madrid, Spain

^5^ Instituto de Ciencia de Materiales de Madrid, Consejo Superior de Investigaciones

Científicas, Spain

^6^ BM25-SpLine (Spanish CRG Beamline) at the European Synchrotron (ESRF), Grenoble, France

^7^ Centre for Cooperative Research on Alternative Energies (CIC energiGUNE), Basque Research and Technology Alliance (BRTA), Vitoria-Gasteiz, Spain

^8^ Sandia National Laboratories, Livermore (CA), USA

INDEX:

1) XRD experimental set-up …2

2) Statistics of structural properties by SEM …2

3) Full spectrum XRD including Co oxalate information …3

4) Changes in the sample morphology induced by

chemical delithiation (by AFM) …4

5) Models of morphology evolution of polycrystalline LCO

during delithiation …5

6) Monoclinic phase discussion …7

1. **XRD experimental set-up**

**
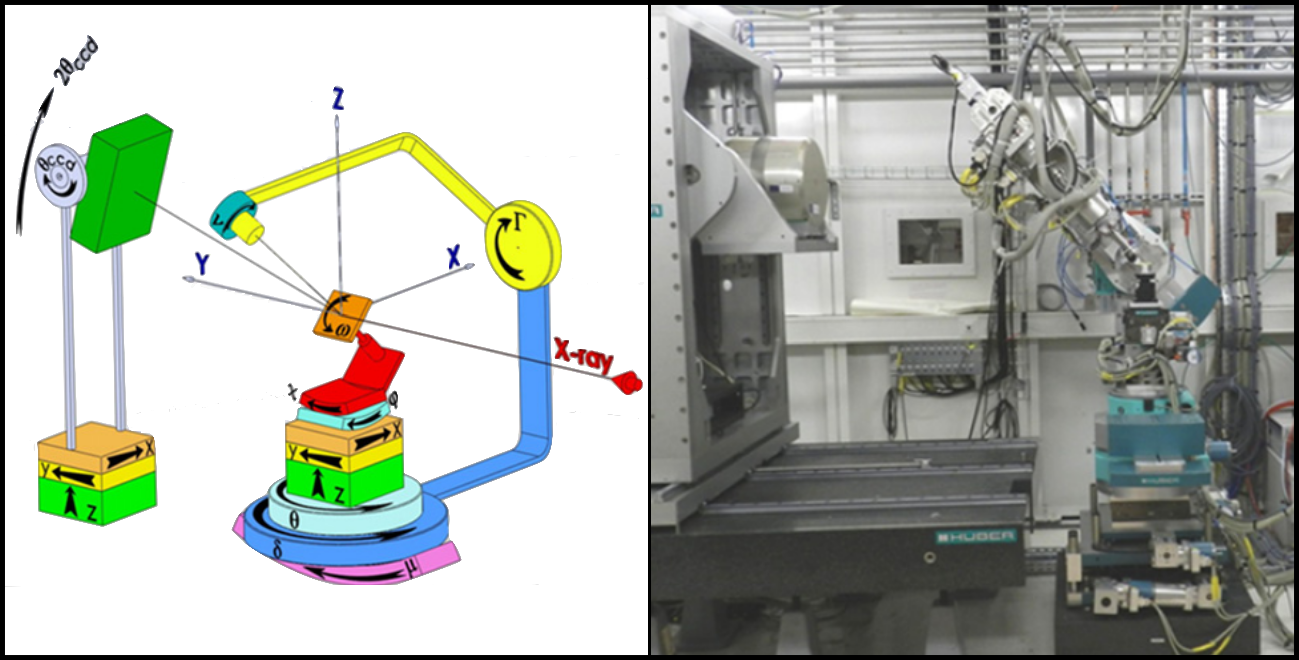
**

**Figure S1.** Left: x-ray diffraction set-up scheme. Right: experimental apparatus. *Reprinted from Nuclear Instruments and Methods in Physics Research Section A: Accelerators, Spectrometers, Detectors and Associated Equipment, 716, J. Rubio-Zuazo, P. Ferrer, A. López, A. Gutiérrez-León, I. da Silva, G.R. Castro, The multipurpose X-ray diffraction end-station of the BM25B-SpLine synchrotron beamline at the ESRF, 23-28, Copyright (2013), with permission from Elsevier.*

1. **Statistics of structural properties by SEM**

**Figure S2.** Statistical analysis of the increase in the size of the surface structures during Regime I (10^‑4^ M). Two lateral correlation lengths are identified and correlated to λ_1_-grain size and λ_2_-size of grain bundle. (a) Radial-averaged self-correlation functions (ACO_iso_) for different exposure times reveal that λ_1_, which is computed from their minima, increases slightly. On the other hand, (b) radial-averaged power spectra densities (PSD_iso_) demonstrate that increase in λ_2_ is higher. The increase is computed from the interceptions of their slopes with the “No correlation”-labelled line, which defines the length scale where any correlation is extinguished. This suggests that the increase in the structure size is due mainly to the grain coalescence into bundles, rather than grain growth.

**Figure S3.** Evolution of the height distribution of the LCO thin film with the exposure conditions. Such distribution is calculated from calibrated SEM images. Symbols plot the experimental data and the curves denote their fit using a Gaussian function (red curve) to estimate the surface contribution and a background (blue curve) that corresponds to the tail coming to the surface of the bulk distribution of the porosity. The data on the ends accumulate the heights outside of the range of the measurements.

1. **Full spectrum XRD including Co oxalate information**


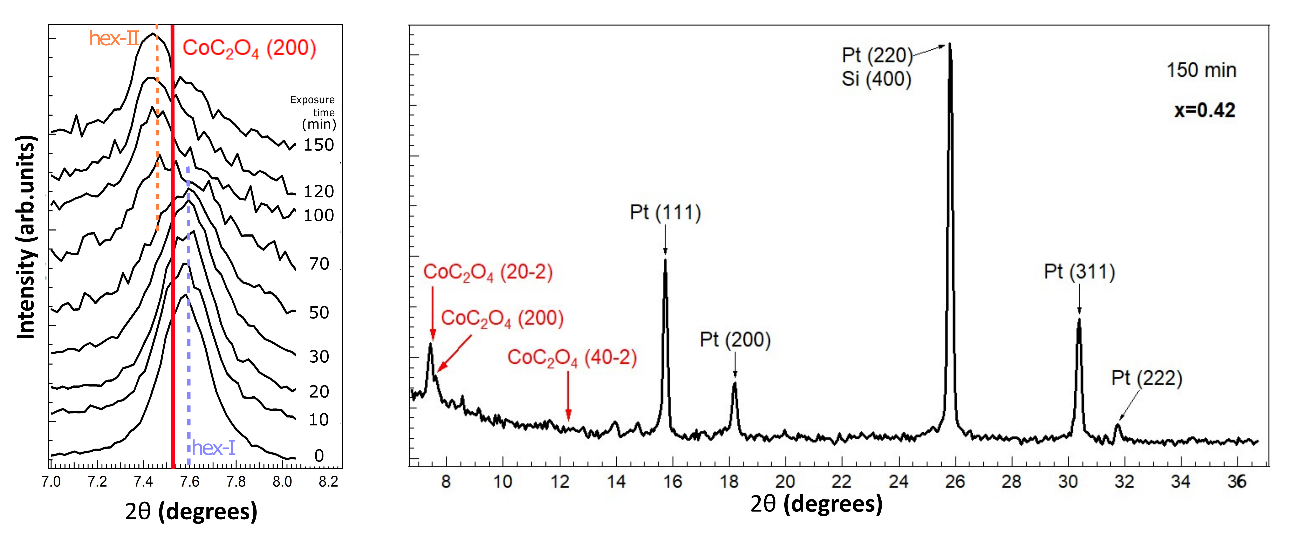


**Figure S4.** XRD spectra of a LCO thin film exposed to oxalic acid. The positions where the most intense cobalt oxalate peaks should appear are marked by red arrows. Peaks (200) and (20-2) coincide with LCO peaks, but the lack of intensity for the (40-2) reflection indicates that there is no cobalt oxalate present in significant amounts.

1. **Changes in the sample morphology induced by chemical delithiation (by AFM)**

We have also performed Atomic Force Microscopy images in several samples. In particular, Figure S5 shows the topography acquired in Amplitude Modulation Dynamic Mode of the two samples analyzed by XPS (Figure 9 in the main text). We can distinguish an increase in the roughness, in good agreement with the SEM analysis, and bundles of grains separated by deep grooves for the case of the delithiated sample.





**Figure S5.** Change in the sample morphology with the chemical delithiation: (left) As-grown LCO film, and (right) 65 min-exposed at 10^-2^ M oxalic acid. Scanned area=2.5 x 2.5 m^2^ in Amplitude Modulation Dynamic Mode. The corresponding RMS roughness is specified at the bottom. *Figure created with WSxM 5.0 Develop (*[*http://www.wsxm.es*](http://www.wsxm.es/download.html)*)^[[1]](#footnote-1)^.*

1. **Models of morphology evolution of polycrystalline LCO during delithiation**

*Regime I. Mild delithiation*

Mild delithiation using diluted oxalic acid (10^-4^…10^-3^ M) and short exposition times extracts Li-ions from the surface shell of the film. This extraction occurs preferably in the surface of the grains, whose lattice can be freely expanded^[[2]](#footnote-2)^ and less in the contact sections (triple junction points—top scheme, Fig. S6a) between neighboring grains, where the compression resulting from the delithiation increases the Li extraction barrier. This results in an inhomogeneous lattice expansion (solid magenta profile in Fig. S6a) that causes a decrease in the dihedral angle $\beta$ (bottom scheme) below the equilibrium value $\beta_{e}$.$\beta_{e}$ defined from the balance between interface tensions along the out-of-plane direction (bottom scheme in Fig. S6a) as:

$2\gamma_{S}cos\left( \beta_{e}/2 \right)=\gamma_{GB}$ (S1),

where $\gamma_{S}$ and $\gamma_{GB}$ are the surface and grain boundary (GB) energy, respectively. A lower $\beta$ (i.e., $\beta<\beta_{e}$) implies the generation of a normal tensile stress $\sigma_{N}$ according to the Laplace-Young equation:

$\sigma_{N}=-\hat{\nabla}_{S}\cdot\left[ 2\gamma_{S}cos\left( \beta/2 \right)-\gamma_{GB} \right]\hat{N}$ (S2),

with $\sigma_{N}⇈\hat{N}$, where $\hat{\nabla}_{S}$ is the surface gradient and $\hat{N}$ is the unit normal vector. The tensile stress produces the zipping/filling of the grooves^[[3]](#footnote-3)^ giving rise to the bundling of the grains into larger structures. The grooves become shallow gaps (as sketched in Fig. S6a, top scheme) inside the structures. The contrast in the SEM images shown in Figs. 2 and S6a (insets) allows us to discern between grooves (black outlines) and shallow gaps. Fig. S6a shows the morphology evolution $h(t)$, where $h(t)$ denotes the surface height, around a groove.

*Regime II. Delithiation by leaching*

Unlike the regime I, leaching induces grooving, since the concentrated oxalic acid erodes preferentially the surface sites with higher curvatures $\kappa$ (and greater reactivity) decreasing the granular volume of the film. As shown in Fig. S6b, these sites correspond to the triple junction points between grains where the erosion rate (upper curves with color-scaled intensity) is enhanced with respect to the homogeneous rate on the surface of the grains. The evolution of the groove morphology resulting from leaching as $\partial_{t}h\left( t \right)\propto-\kappa\approx\nabla_{S}^{2}h(t)$ is outlined in Fig. S6b.


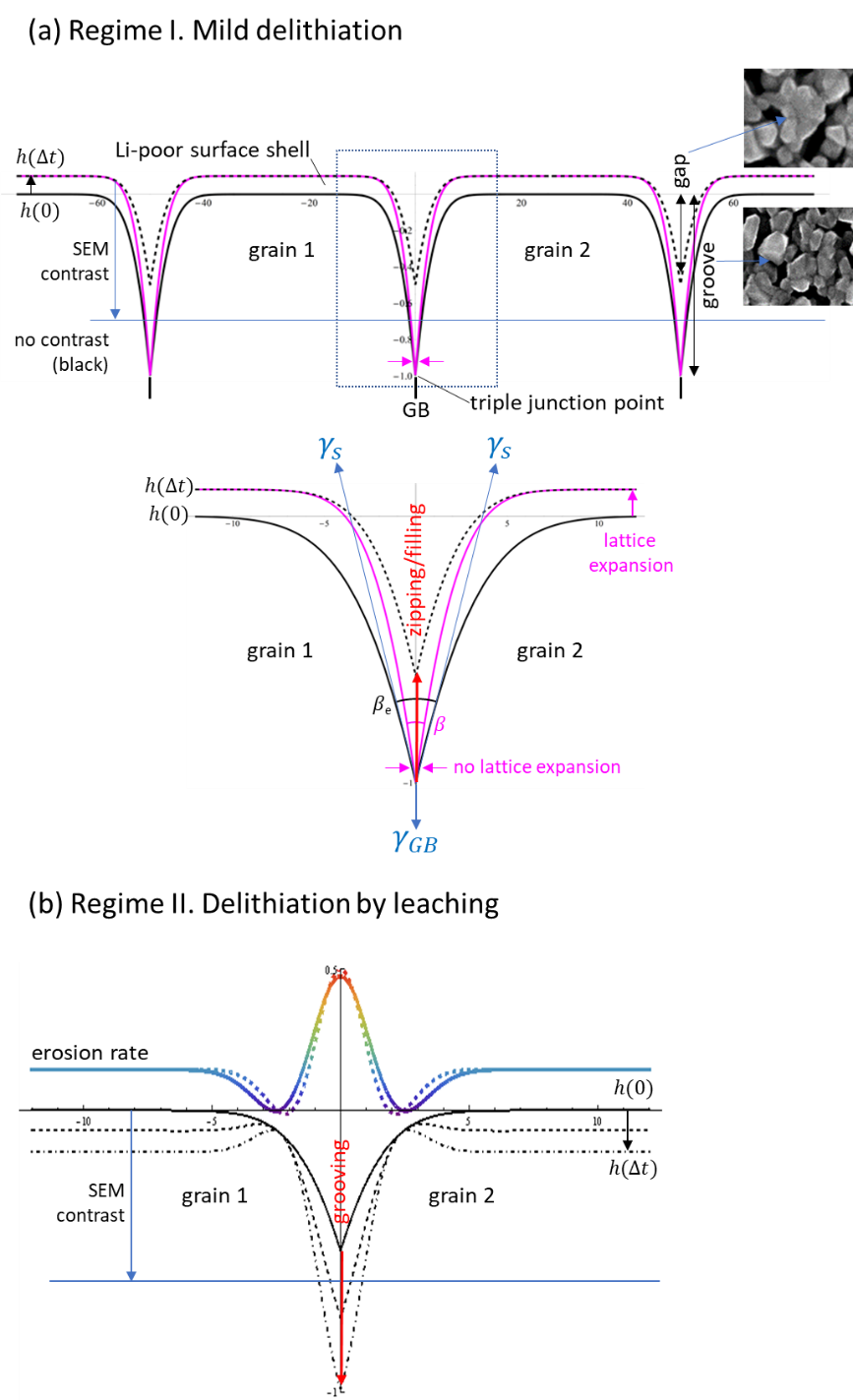


**Figure S6.** Morphology evolution around the groove where a GB emerges to the surface during different delithiation regimes: (a) regime I—mild delithiation and (b) regime II—delithiation by leaching. The progression of morphology profiles $h(t)$ is solid->dashed->dotted curves. The magenta profile in (a) shows the inhomogeneous lattice expansion, while the color-scaled profiles in (b) plot the leaching erosion rate, which is $\propto\kappa$ (surface curvature). The bottom scheme in (a) depicts the out-of-plane balance of involved interfaces tensions. The SEM contrast is used to discern between grooves and inner shallow gaps. *Figure created with Mathematica 12.0 Wolfram (*[*https://www.wolfram.com/mathematica*](https://www.wolfram.com/mathematica/)*).*

1. **Monoclinic phase discussion**

**
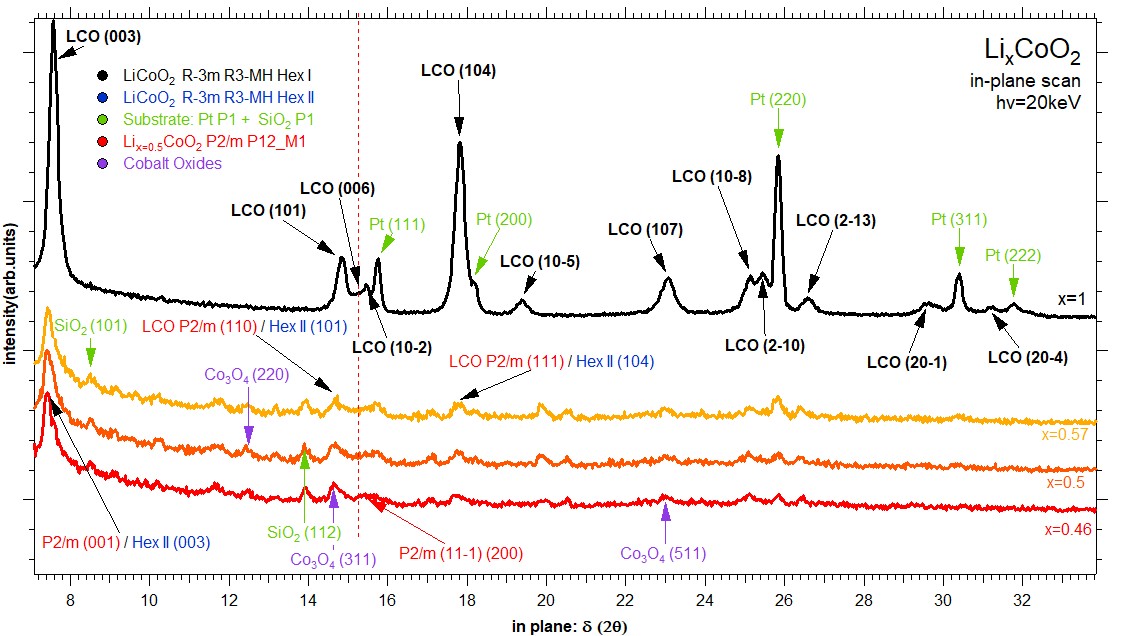
**

**Figure S7.** X-ray powder diffraction spectra corresponding to the as-grown LCO structure (top, black line) and to delithiated Li_x_CoO_2_ samples corresponding (colored lines, from top to bottom) to x=0.57 (light orange line), x=0.5 (orange line) and x=0.46 (red line). The expected location of the main x-ray reflections for the hexagonal phases, the monoclinic phase and cobalt oxides are highlighted. Residual reflections from the Pt layer underneath the LCO film and the Si substrate are detected.

Figure S7 shows the peak identification for possible LCO structural phases due to the chemical delithiation process and Li deintercalation and the substrate . Notice that the X-ray reflections for LCO Hex I, LCO Hex II and LCO monoclinic are very close to each other and are difficult to identify within the full angular range probed. This is due to the fact that the monoclinic phase is due to a minor distortion of the Hex II phase. We must add to this problem the fact that, as explained in the main text, the delithiation is inhomogeneous, having different x values in small regions on the sample.

In Figure S7, the x=1 spectra in black is compared to spectra with x close to 0.5, expecting the monoclinic phase to appear for x=0.5. Monoclinic peaks are labelled P2/m. In most cases, they coincide with hexagonal peaks. The main expected difference would be an intensity decrease and disappearance of the (006) reflection, but this reflection is hardly seen in the pristine sample, due to the proximity of the Pt(111) reflection. This was circumvented using the optical alignment of the XRD data in order to decrease the relative intensity of the Pt peaks, as can be seen in the spectra for the delithiated sample in Fig. S7. However, no clear observation of any change in the (006) reflection was observed due to the proximity of other peaks, as LCO(10-2). The shift of the Hex I and Hex II reflections with Li contents also hinders the identification of the origin of other possible monoclinic peaks. With these data, we can neither conclude the observation of the monoclinic phase nor rule out this possibility.

Some extra peaks observed that do not correspond to the different LCO phases come from the Pt and Si substrate. Their visibility and relative intensity depends on the optical alignment of the sample and probably on the quality and degradation of the sample surface.

1. I. Horcas, R. Fernández, J. M. Gómez-Rodríguez, J. Colchero, J. Gómez-Herrero, and A. M. Baro. WSXM: A software for scanning probe microscopy and a tool for nanotechnology. *Rev. Sci. Instrum.* **78**, 013705 (2007). [↑](#footnote-ref-1)
2. G. G. Amatucci, J.M. Tarascon, L. Klein. CoO2, The End Member of the Lix CoO2 Solid Solution. *J. Electrochem. Soc.* **143**, 1114 (1996). [↑](#footnote-ref-2)
3. A. González-González, G. M. Alonzo-Medina, A. I. Oliva, C. Polop, J. L. Sacedón, and E. Vasco, Morphology evolution of thermally annealed polycrystalline thin films. *Phys. Review B* **84**, 155450 (2011) [↑](#footnote-ref-3)
